# Evaluation of dosimetric and spatial accuracy of a virtual cone technique for radiosurgery using linac‐integrated CBCT‐based polymer gel dosimetry

**DOI:** 10.1002/acm2.70081

**Published:** 2025-03-18

**Authors:** Tenzin Kunkyab, Michael Lamey, Andrew Jirasek, Michael Kudla, Nathan Becker, Benjamin Mou, Derek Hyde

**Affiliations:** ^1^ Department of Computer Science, Mathematics, Physics and Statistics University of British Columbia Okanagan Kelowna British Columbia Canada; ^2^ Department of Medical Physics BC Cancer‐Kelowna Kelowna Canada; ^3^ Department of Medical Physics The Ottawa Hospital Ottawa Ontario Canada; ^4^ Department of Surgery The University of British Columbia Vancouver British Columbia Canada

**Keywords:** gel dosimetry, stereotactic radiosurgery, virtual cone

## Abstract

**Purpose:**

This study evaluates the dosimetric and geometric precision of a virtual cone technique using CBCT‐based polymer gel dosimetry, enabling radiation delivery, and imaging readout within an identical spatial coordinate system.

**Methods:**

We created a C# script for a virtual cone technique that generates a treatment plan with 10 gantry arcs at 0°, 36°, 72°, 288°, and 324° couch angles, with 2 arcs per couch angle using 45° and 135° collimator angles. Two verification plans using Eclipse v15.6 (AcurosXB) were created with 20 Gy at the maximum dose for: (1) a cylindrical gel, with an additional calibration region; (2) a 3D printed anthropomorphic skull phantom with a gel insert. The 50% isodose (10 Gy) width through the central axis of the axial and sagittal planes (SPs) were measured for the gel experiment. The distance between the centers‐of‐masses of the 10 Gy isodose region of the plan and the gel (skull phantom) were calculated for an end‐to‐end spatial accuracy test.

**Results:**

The maximum point dose measured with gel was within 1% of the plan, though the gel measured 50% isodose widths of 5.56±0.02 mm, 5.65 ±0.04 mm, 4.23 ±0.01 mm for axial (anterior–posterior), axial (left–right), sagittal (superior–inferior) respectively, which were slightly narrower than Eclipse (1.29 mm maximum difference in the SP due to CBCT slice thickness). The center‐of‐mass distance was 0.66 mm for the gel experiment, and 0.94 mm for complete end‐to‐end testing with the anthropomorphic phantom, including CBCT setup (kV‐MV isocenter uncertainty).

**Conclusion:**

The 50% isodose width of the gel measurement was 5.15 mm (mean), which was tighter than our Eclipse v15.6 beam model. The end‐to‐end spatial accuracy test, only achievable with gel dosimetry using CBCT readout, resulted in sub‐millimeter accuracy. This study demonstrates the value of gel dosimetry in verifying the dosimetric and spatial accuracy of this high precision, stereotactic technique.

## 1 INTRODUCTION

Trigeminal neuralgia (TN) is a rare neurological condition that affects 4 to 13 people in 100 000 annually.^1^, ^2^, ^3^, ^4^, ^5^ A systematic review reported a prevalence rate of 12.6 to 28.9 per 100 000 patients annually.^6^ Although the incidence of TN is uncommon, it is among one of the most common facial pain syndromes.^1^, ^3^, ^7^ TN is characterized by severe, unilateral, and episodic pain that has significant implications on the patient's quality of life.^8^, ^9^, ^10^, ^11^ Treatments for TN include medications, surgical procedures, and noninvasive radiotherapy utilizing stereotactic radiosurgery (SRS).^7^

Traditionally, TN was treated with SRS utilizing a Gamma Knife unit, a Cyberknife or C‐arm Linac with a physical stereotactic cone.^5^, ^7^, ^12^, ^13^, ^14^, ^15^, ^16^ A Gamma Knife or Cyberknife unit is not readily accessible in many radiotherapy centers. A C‐arm Linac with a physical stereotactic cone has its own limitations, including the requirement for additional machine quality assurance, a cone‐specific dose calculation algorithm, and patient‐specific quality assurance, which significantly increases the clinical workload.^17^, ^18^ To mitigate these challenges, researchers at the University of Alabama at Birmingham (UAB) developed a virtual cone technique utilizing a high‐definition multi‐leaf collimator (HDMLC) from Varian (Varian Medical Systems, Palo Alto, CA) to deliver a small spherical dose distribution with 70–90 Gy at the isocenter.^16^ The isocenter is typically placed such that the 50% isodose volume (40 Gy) is close to the brainstem, which is a critical structure inside the brain.^19^ The dose limit for the brainstem for an SRS radiotherapy is 15 Gy.^20^ Thus, an evaluation of the dosimetric and geometric accuracy of the virtual cone delivery is of high importance to overcome serious complications, for instance, brainstem necrosis that can result in death or disabling neuropathy.^21^ This virtual cone method produces a spherical dose distribution that approximates that of a 5 mm physical stereotactic cone. A static field is defined by the two central collimator leaf pairs and collimator angles of 45 and 315 degrees are used to deliver an X pattern with two arcs at each couch angle. A 10‐MV flattening filter free (FFF) beam is used to deliver 10 arcs, and the monitor units (MU) delivered per degree is varied by the sine function of the gantry angle in order to produce the spherical dose distribution. The use of the 10‐MV FFF beam with a maximum dose rate of 2400MU/min enables the treatment time to be reduced from a 6‐MV FFF beam with a maximum dose rate of 1400MU/min.^22^

Accurate dosimetry of the virtual cone technique is challenging due to the small field conditions that are well‐known in SRS radiotherapy: the lack of lateral charged particle equilibrium in beam axis, partial blockage of the primary photon source and detector size larger than the photon beam dimensions.^23^, ^24^, ^25^ A detector size with sub‐millimeter dimension such as cc13 ion chamber (IBA Dosimetry Group, Herndon, Virginia, USA), commonly used in our clinic, underestimates the dose due to its low mass density, whereas, over responsive characteristics are observed in other detectors such as MicroDiamond (PTW, Freiburg, Germany), made with high mass density material.^26^ Gafchromic film series (EBT3/EBT‐XD, Ashland Specialty Ingredients, Bridgewater, NJ, USA) are typically the gold standard for SRS radiotherapy technique, due to its high spatial and dosimetric resolution, however, the film scanning and subsequent processing inherently removes the capability to measure an end‐to‐end spatial accuracy at the treatment position/setup. A dosimeter that can measure both the dosimetric and geometric precision accurately and simultaneously is clinically useful. Furthermore, a dosimeter that can perform a complete end‐to‐end spatial accuracy evaluation in 3D space is highly beneficial to the clinical workflow for this type of high precision SRS virtual cone technique.

3D Polymer gel dosimeters are fabricated using radiation sensitive chemicals that undergo polymerization and crosslinking when exposed to ionizing radiation. Gel dosimeters offer distinct advantages over traditional 1D and 2D dosimetry for treatment verification of advanced radiation therapy procedures, especially when steep dose gradients exist.^27^ The intrinsic 3D characteristics of the gel dosimeter enables a dose distribution to be mapped for the entire three‐dimensional space. Gel dosimetry eliminates the need to utilize multiple point dosimeters (e.g., Thermoluminescent dosimeter) or to measure the dose in multiple planes (e.g., film), if 3D verification is required. Gels are tissue equivalent, have high spatial resolution, low energy dependence,^28^, ^29^ and can be incorporated into an anthropomorphic phantom.^30^ As such, polymer gel dosimetry is useful for end‐to‐end testing of complex radiotherapy techniques.^31^ Despite these potential benefits, clinical adoption of gel dosimeters is still relatively uncommon and has potential for further development.

Polymer gel dosimetry relies on an imaging tool to read out the 3D dosimetric data. The imaging tool may be magnetic resonance imaging (MRI), x‐ray computed tomography (CT), optical CT, or Linac‐integrated kilovolt cone beam CT (CBCT).^27^, ^32^, ^33^, ^34^, ^35^, ^36^ MRI offers excellent spatial and contrast resolution, however, MRI is not readily accessible for many radiotherapy centers. x‐Ray CT and optical CT are generally more accessible, however, moving the dosimeter to another system for the readout introduces an inherent spatial inaccuracy (also holds true for MRI imaging). Furthermore, the differences in the spatial frame of reference between the Linac and the CT can complicate the dose registration.^37^ CBCT has been shown to have comparable accuracy and precision to CT acquisition (the gold‐standard) for the clinically relevant intracranial stereotactic space.^38^ For treatment verification of complex radiotherapy techniques such as SRS, a readout method based on Linac‐integrated CBCT is appealing for multiple reasons. It provides unmatched spatial accuracy over the other imaging modalities since the setup for treatment position is identical to the readout position. Therefore, there is no user setup error associated with the dose delivery and measurement.^35^, ^37^ Second, after the radiation is delivered to a gel dosimeter the readout can be acquired in the same session, as a wait time of 20 min, for polymerization and crosslinking, is adequate (> 90% polymerization yield).^35^ The use of CBCT modality is associated with limitations such as a reduced signal‐to‐noise ratio and the presence of imaging artifacts. However, various image processing techniques, including background subtraction, adaptive mean filtering, and remnant artifact removal, are employed to mitigate these issues and improve image quality.^35^, ^36^, ^39^, ^40^ A previous study by Pant et al. has used gel dosimetry with CBCT imaging to verify the treatment isocenter of the Linac, and quantify the spatial uncertainty associated with it.^41^ Moreover, Adamson et al. performed a proof‐of‐principle study to visualize the 3D dose distribution on the gel dosimeter immediately after irradiation, using CBCT imaging.^42^ A recently published study investigated the reproducibility of CBCT based gel dosimetry workflow in a dual‐institution setting.^36^ However, the clinical applications of Linac‐integrated CBCT in gel readout is still not as routine as other imaging modalities, such as MRI and x‐ray CT.

In this work, we investigated the feasibility of a polymer gel dosimetry workflow with CBCT readout to verify the dosimetric and complete end‐to‐end spatial accuracy of the virtual cone technique in our clinic.^16^ For the end‐to‐end spatial accuracy test, we utilized a low‐cost 3D printed anthropomorphic skull phantom for treatment planning and virtual cone treatment delivery.

## 2 MATERIAL AND METHODS

### 2.1 Implementation of the virtual cone technique

We developed an in‐house Eclipse executable script in C sharp to generate a virtual cone treatment plan, based on the original work by the UAB.^16^ As in their work, our Eclipse script generated 10 arcs for five couch positions: 2 arcs per each unique couch angle. The five couch angles were 0°, 36°, 72°, 288°, 324° degrees with the collimator set to 45° and 315° for each couch position. For 0° couch angle, a 360° arc was used, whereas the remaining couch angles had 180° partial arcs.^16^ The original UAB study recommended the formula: 2.1 +0.36 mm‐dosimetric leaf gap (DLG) to obtain the MLC leaf separation to account for the institutional differences in DLG settings.^16^ As such, our institutional DLG of 1 mm, resulted in 1.4 mm leaf separation for the virtual cone technique, and this separation was utilized throughout this study.

### 2.2 Treatment planning and delivery

As per the UAB technique, the MU per degree varied proportionally to the sine of the gantry angle.^16^ The dose calculation algorithm used was Acuros XB (Varian Medical Systems, Palo Alto, CA) and all the treatment plans were delivered with a Varian Truebeam (Varian Medical Systems, Palo Alto, CA) with all the Edge capabilities (HDMLC, 6DOF couch, Identify SGRT). The 10 MV FFF beam with 2400 MU/min dose rate was used for all the experiments. Note that we did not implement a new beam model specifically for this virtual cone technique, but utilized our clinical beam model in which the DLG had been optimized for stereotactic ablative radiotherapy treatments. Although it is beyond the scope of the current study, we performed preliminary testing with ion chamber, diamond detector, and EBT‐XD film (Ashland Chemical, Covington, KY) to verify that the model was still reasonable for virtual cone technique prior to this gel dosimetry study.

The clinical treatment plan originally with a prescription dose of 80 Gy at isocenter (using Eclipse v15.6) were recalculated on the respective quality assurance (QA) phantom with a maximum dose of 20 Gy to prevent gel saturation. Two phantom experiments were conducted: (1) 3D Gel with an additional calibration region (2) Gel inserted into a 3D printed anthropomorphic phantom for end‐to‐end spatial accuracy test. For all the verification plans, a dose grid size of 1 mm was utilized in this study.

**FIGURE 1:**
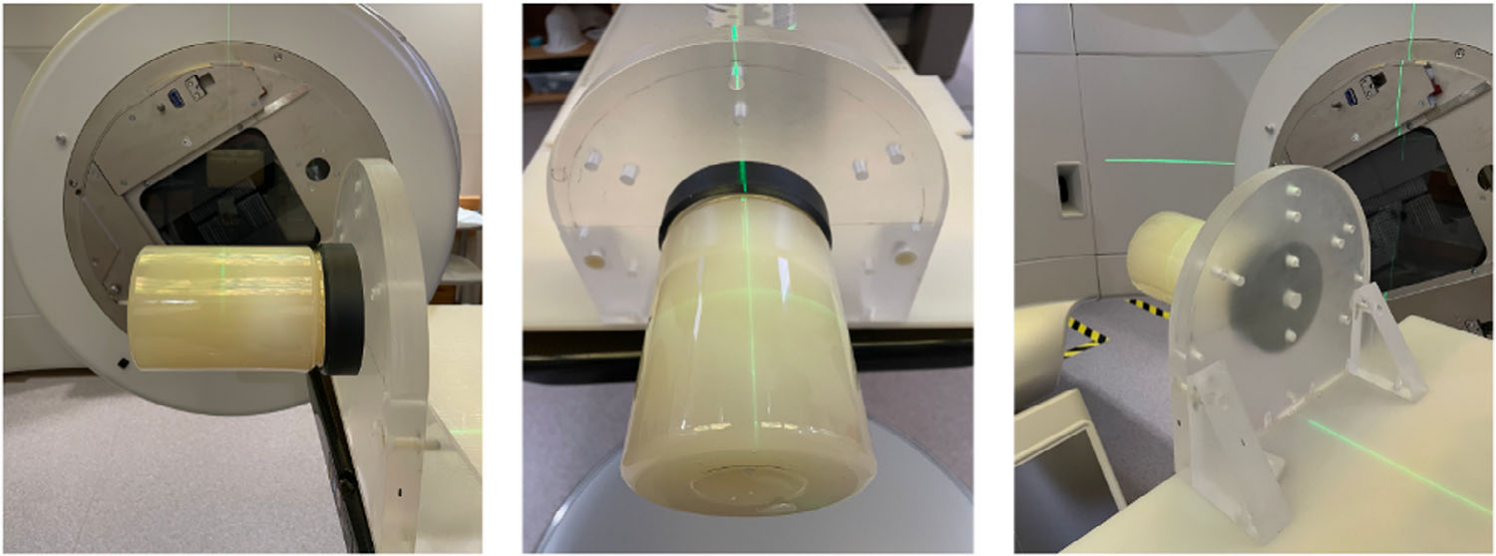
All panels illustrate the gel experiment set‐up for the dosimetric analysis containing a verification plan with virtual cone and calibration region is shown from different angles.

The goal of our first gel experiment was to characterize the relative dosimetric properties of the gel dosimeter. Therefore, for the dosimetric analysis, we used the gel only experiment with the verification plan that contains a virtual cone and a calibration region on the same gel. The calibration plan involved a (– 4 cm) longitudinal couch shift and was delivered on the same gel with 2 co‐planar arcs with a static MLC. The same field size was used for the calibration plan as the virtual cone technique and 10 MV FFF beam energy was used to deliver a maximum dose of 20.1 Gy in the v15.6 verification plan. To ensure that the calibration dose region does not affect the virtual cone dose, a rigorous dose profile analysis was performed in the treatment planning system prior to treatment delivery. The choice of calibration dose being slightly higher than maximum verification dose was to ensure that the entire dose distribution of virtual cone is comfortably covered within the calibration region (Appendix A). A single gel was used for the calibration process to avoid any potential differences due to the chemical batches and intra‐user manufacturing variability. The setup for this irradiation is shown in Figure 1.

**FIGURE 2:**
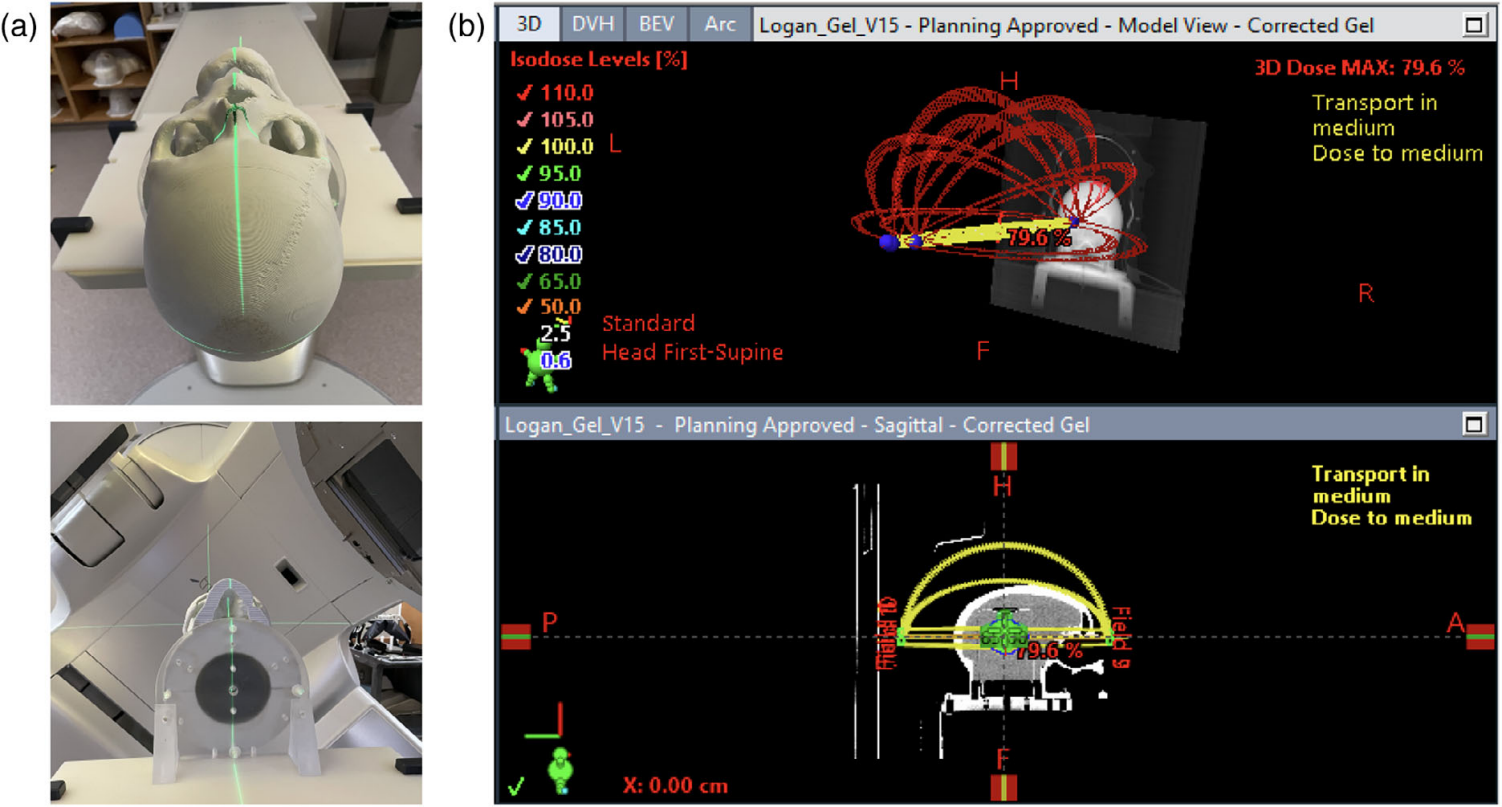
(a) Shows the 3D printed anthropomorphic skull phantom setup. (b) The virtual cone plan generated on the planning CT of the skull phantom is shown.

The verification plan generated for the 3D printed anthropomorphic skull phantom was used for a dedicated end‐to‐end spatial accuracy verification. The skull phantom was setup using CBCT and treated like an actual TN patient with the virtual cone technique. The setup for this radiation delivery is shown in Figure 2a and the virtual cone plan on the skull phantom is presented in Figure 2b.

### 2.3 Gel dosimetry workflow

#### 2.3.1 Gel fabrication

A NIPAM gel dosimeter was fabricated based on a previously published protocol.^43^ In summary, the dosimeter composed of 15% N‐isopropylacrylamide (NIPAM, Sigma Aldrich, Mississauga, ON, Canada), 4.5% N,N’‐methylenebisacrylamide (BIS or MBA, Sigma), 5% 300 bloom gelatin (Sigma), 10 mM tetrakis (hydroxymethyl) phosphonium chloride (THPC, Sigma), and 75.5% deionized water. After carefully following the mixing procedure, the dosimeter was cooled in a 1 L jar (Modus Medical, London, ON, Canada), in a water bath inside a refrigerator for at least 5 h. Prior to the irradiation, the gel dosimeters were allowed to stabilize at the room temperature for at least 5 min.

#### 2.3.2 3D printed anthropomorphic skull phantom

A stereolithography (STL) model required for the 3D printing process, was acquired from a patient CT image from the The Cancer Imaging Archive image database.^44^, ^45^ The skull was segmented using an Hounsfield unit (HU) threshold setting (500 (lower)–1200 (higher)) and the file was exported in an STL format using the 3D slicer software.^46^ The HU threshold range was chosen visually such that an accurate representation of the skull structure was obtained from the 3D slicer software. A 3D modeling software, Meshmixer (Autodesk, San Francisco, USA), was used to clean and re‐model the skull phantom, such that the gel jar can be inserted into the anthropomorphic phantom. The outer skull structure was 3D printed with a CaCO3 polylactic acid (PLA) (Protopasta, Vancouver, Washington, USA) using a 0.6 mm nozzle, bed 60°C, chamber 40°C.^47^ The CT number of the skull structure (CaCO3) was 636 HU and had a physical density of 1.45 g/cm^3^. We selected the CaCO_3_ PLA material as a representation of bone over other high‐density PLA used in a previous study, such as iron, copper, and bronze,^48^ in order to reduce artefacts in our background and postirradiation CBCT images. Image distortion and artefact (streaking) is commonly observed in images of the previously used high‐density materials, and would be detrimental to the gel image analysis.^48^ The ‘brain’ was represented with VytaFlex‐50, a urethane rubber casting material (Smooth‐On Inc, Macungie, Pennsylvania, USA), with a CT number 30 HU and a physical density of 1.04 g/cm^3^. The Acuros XB calculation with dose‐to‐medium was used, both the CaCO3 and VytaFlex‐50 materials were assigned as water with their appropriate mass densities overridden for dose calculation.

#### 2.3.3 Gel readout and analysis

Six preirradiation CBCT and six postirradiation CBCT images were acquired in order to reduce image noise and provide background subtraction. Note that as per the prior study, a 20 min wait time was allocated before imaging the gel after the irradiation.^49^ For both the gel experiments, the six preirradiation and six postirradiation CBCT images were individually averaged, and then background subtracted from one another. An adaptive mean filtering (3 × 3 kernel) and remnant artefact removal (window span = 7) was applied to the background subtracted image slices to reduce the image noise and artefacts, respectively.^39^, ^40^

For 3D gel dosimetric study, the imaging parameters used for both the pre‐ and post‐scans was a slow CBCT scan mode with 1620 mAs, 125 kVp, 5400 projections, 0.5 mm pixel size, and 1 mm slice thickness. The calibration region, delivered with two co‐planar arcs, was used to acquire a calibration curve relating the dose to the CT number. The calibration curve (Equation 1, Appendix A; Figure A1) obtained was used to calibrate the irradiated gel region containing the virtual cone dose distribution. To enable pixel by pixel calibration without an interpolation step, we recalculated the delivered verification plan on one of the acquired CBCT images. In the recalculation of verification plan on the CBCT image, gel dosimeter was assigned as “water” and the mass density of the gel (1.025 g/cm^3^)^50^ was overridden as done in the prior work.^36^ The recalculation was performed in our workflow to eliminate image/dose registration during the analysis stage. Finally, we measured the 10 Gy isodose width in axial and sagittal planes (SPs) of the verification plan and the gel data.

For the end‐to‐end spatial accuracy analysis with the anthropomorphic skull phantom, an iterative CBCT scan mode with 2160 mAs, 125 kVp, 5400 projections, 0.5 mm pixel size, and 1 mm slice thickness resolution was used. We increased the milliampere‐seconds and used the iterative reconstruction method since the gel was imaged within the anthropomorphic phantom. This was done to maintain the geometric position and eliminate any user‐set up error from the removal of the skull phantom. Using a higher milliampere‐seconds and iterative reconstruction reduce the image noise caused by the heterogeneous materials in the skull phantom, while not contributing significantly to the image distortion. Moreover, the role of image processing (image averaging, background subtraction, adaptive mean filter, and remnant artefact removal) in our gel workflow was necessary to recover the underlying signal of the dose distribution in the noisy image of the anthropomorphic phantom.

For end‐to‐end spatial accuracy test, a self‐calibration technique was performed by selecting the central slice of the virtual cone verification plan and calibrated the entire gel data with the corresponding iterative CBCT slice, in (Appendix A; Figure A1).^36^ Note that a single slice from the verification region was used to calibrate the entire region, since the primary goal of the end‐to‐end testing is to only acquire the spatial accuracy information. To enable pixel‐by‐pixel calibration in the phantom study, the original planning CT was interpolated to the dimensions of the CBCT in order to obtain the calibration curve parameters. The 3D center of mass (COM) of the 10 Gy isodose region was calculated for the Eclipse verification plan and the gel, and then the Euclidean distance between them was then calculated. The experimental set‐up and verification plan involving 3D anthropomorphic phantom is shown in Figure 2.

The entire gel analysis was completed using an in‐house developed MATLAB script (MathWorks, Natick, USA). For the COM calculation, *regionprops3*, a built‐in function in MATLAB was used.^51^, ^52^ Moreover, the equivalent diameter of the spherical 50% isodose region in millimeter was also measured for 3D anthropomorphic skull phantom study resembling an actual virtual cone treatment delivery.

The empirical calibration curve model is as follows:

(1) $$\Delta N_{CBCT}=\alpha +\beta \tanh\left(\gamma D-\varphi \right)$$

Where $\Delta N_{CBCT}$ is the change in CBCT number of the gel, *D* represents dose in Gy, α,β,γ,φ represents the parameters of the calibration equation. The parameters defining the calibration equations, acquired from the two gel experiments with 95% confidence interval are provided in Appendix B (Table B1).

## 3 RESULTS

### 3.1 3D Gel dosimetric analysis

**FIGURE 3:**
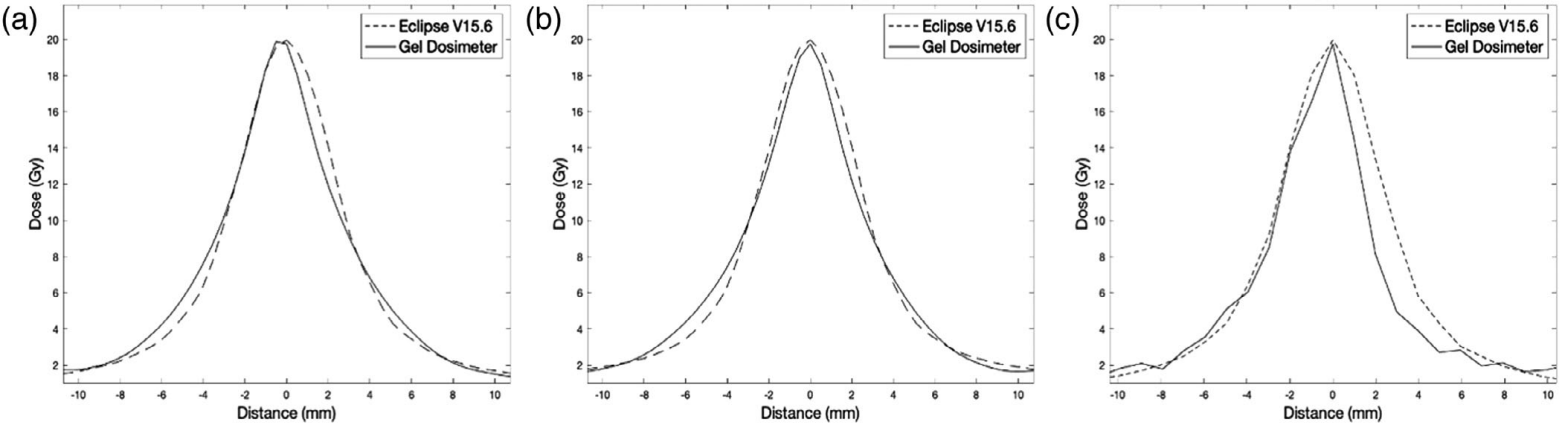
(a) Illustrates the dose profile along the (a) axial plane (anterior–posterior), (b) axial plane (left–right), (c) SP (superior–inferior) using the 3D gel dosimetric analysis. SP, sagittal plane.

Figure 3 presents the dose profile along the axial plane (anterior–posterior), axial plane (left–right) and SP (superior–inferior) through the pixel containing the maximum dose of the Eclipse verification plan and the spatially corresponding pixel position in the gel image. The maximum peak dose through the axial plane (LR) profile is observed to be 1% smaller than the verification plan. The percent difference between the maximum dose in axial plane (AP) and SP is 0.5% and 0.9%, respectively. In the case of SP, we observe that the profile along this direction appears to be truncated. We calculated the Euclidean distance between the two centers of masses (3D) of the 10 Gy isodose region of the verification plan and the gel, resulting in measured distance of 0.66 mm between the two centers of masses. However, this does not represent a complete end‐to‐end spatial accuracy test since the verification plan was re‐calculated on one of the acquired CBCT image prior to the analysis to match the plan image and CBCT image dimensions for analysis purposes. The 0.66 mm COM distance between the plan and the gel represents the intrinsic and baseline spatial accuracy achievable with this type of dosimetry workflow.

Table 1 presents the 10 Gy isodose width of the profiles across AP, axial plane (LR), and SP. The largest deviation between the Eclipse verification plan and gel was in the SP of about 1.29 mm. In Figure 3c, we observed a partial volume effect due to the CBCT resolution in the slice thickness direction. In axial plane (LR and AP), a maximum deviation of 0.19 mm is observed by measuring the 50% isodose width between the verification plan and gel, presented in Table 1.

**TABLE 1:** Presents the 50% isodose width of the profile across axial plane (anterior–posterior), axial plane (left–right), and sagittal plane (superior–inferior) using the 3D gel dosimetric analysis.

	Axial plane (Ant‐Post), (mm)	Axial plane (Left‐Right), (mm)	Sagittal plane (Sup‐Inf), (mm)
Calculation (Eclipse v15.6)	5.75 mm	5.73 mm	5.52 mm
Measurement (Gel)	5.56 ± 0.02 mm	5.65 ± 0.04 mm	4.23 ± 0.01 mm

### 3.2 End‐to‐End spatial accuracy test with 3D anthropomorphic phantom and image‐guided radiotherapy

We calculated the Euclidean distance between the two 3D COMs of the Eclipse verification plan and gel. The distance between the two COMs measures 0.94 mm using the 50% isodose region. This investigation represents a complete end‐to‐end spatial accuracy testing of our virtual cone radiotherapy workflow using a low‐cost 3D printed anthropomorphic phantom.

We present the equivalent diameter of the 10 Gy isodose region in millimeters in Table 2. Note that the equivalent diameter of Eclipse v15.6 plan for the end‐to‐end spatial accuracy analysis is calculated using the interpolated CT image to match the CBCT image dimensions for calibration purposes.

**TABLE 2:** Equivalent diameter of the 10 Gy isodose region in the 3D printed anthropomorphic gel experiment in millimeter.

	Eclipse v15.6 (Equivalent diameter of 10 Gy isodose volume in mm)	Gel (Equivalent diameter of 10 Gy isodose volume in mm)
End‐to‐end spatial accuracy	5.41 mm	4.92 ± 0.02 mm

**FIGURE 4:**
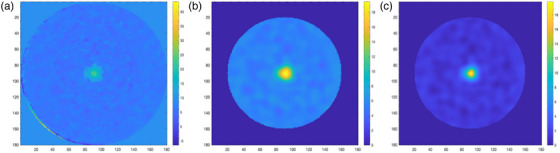
(a) Gel image after averaging and background subtraction. (b) Filtered image with image processing. (c) The final calibrated image.

Figure 4 (a) shows a central slice of the image acquired with iterative CBCT, (b) gel with image processing, (c) and the final calibrated gel slice. We analyzed the dose contribution of CBCT imaging to the gel dosimeter in both of our experiments. In our analysis, for each CBCT scan, a dose of about 0.032 Gy (for 12 scans ∼0.38 Gy) was measured. For our iterative CBCT scan, each scan contributed a dose of 0.043 Gy (for 12 scans ∼0.52 Gy). A total dose percentage of 1.9% and 2.6% were contributed to the gel in dosimetric analysis and end‐to‐end spatial accuracy experiment, respectively. The dose contribution of CBCT readout is significantly lower than the dose delivered, moreover, the use of background subtraction reduces the dose contribution of the CBCT scans during the analysis.

## 4 DISCUSSION

The publication by UAB utilized a stereotactic end‐to‐end verification anthropomorphic skull phantom (Computerized Imaging Reference Systems, Norfolk, VA) with the EBT‐XD GafChromic film dosimeter insert (Ashland Chemical, Covington, KY). An output constancy check was performed with a 2 cm parallel plate ion chamber (Exradin A11, Standard Imaging, Madison, WI). Their treatment plan was normalized to deliver 20 Gy at the isocenter. In their work, the 50% isodose width of a 2.1 mm DLG corresponded to the width of a 5 mm physical stereotactic cone.^16^ More recently, a study by Brown et al. at the Tri‐cities Cancer Center (TCCC) reproduced this SRS technique in collaboration with UAB, and their commissioning data was benchmarked against the previous UAB study.^22^ In their work, EBT3 film (Ashland Advanced Materials, Bridgewater, NJ, USA) and Exradin W2 scintillation detector (Standard Imaging, Middleton, WI, USA) were used to commission this SRS technique with the multi‐leaf collimator leaf gap measuring 2.1 mm. The original UAB study performed the treatment with 10 MV FFF (anisotropic analytical algorithm (AAA), Eclipse version 13.6), whereas the TCCC study used 6 MV FFF beam (Eclipse version 15.6). More recently, Neupane et al. implemented a fixed MLC field setting (0.5 cm by 0.5 cm), using this SRS technique in their center.^53^ This group used SRS MapCHECK (Sun Nuclear Corporation, FL) to measure the 50% isodose width of the dose distribution and reported that the results are comparable to a physical stereotactic cone.^53^ Their study corroborated the previous findings and concluded that a virtual cone technique is indeed a viable method for an SRS treatment for TN. Another recent study by Eric Lobb.^54^ adapted this virtual cone technique to a standard MLC with 5 mm leaf width and concluded that it is feasible to efficiently generate a spherical dose distribution for small brain metastasis. Although, physical stereotactic cones were able to achieve superior peak dose gradients as opposed to the adapted virtual cone technique, the current novel SRS technique could be useful as an alternative method to decrease the cost, complexity, and improve technology consolidation in an SRS program. The virtual cone technique developed by UAB was effectively utilized to treat TN patients in their center.^16^ This relatively novel technique could potentially be replicated in any institution with a Truebeam linear accelerator with SRS capability. Therefore, our current work describes one such instance of replication of the technique at another independent center. Note that each individual institution is fully responsible for implementing the virtual cone technique and conducting its own commissioning measurements.

In our work, we investigated the feasibility of our gel dosimetry workflow to evaluate the dosimetric and spatial accuracy verification of the virtual cone technique planned with the Eclipse v15.6 (Acuros XB). For the dosimetric analysis, we employed a calibration region in the gel dosimeter to obtain the dosimetric calibration curve. The 10 Gy isodose width profiles showed relatively good agreement between the gel and the Eclipse v15.6. The largest deviation of 1.29 mm, between the plan and gel is observed in SP. This may be caused by the partial volume effect evidently observed as a result of the poor resolution of CBCT in slice thickness (1 mm) direction compared to the axial resolution (0.5 mm) in Figure 3c. Despite this shortcoming, the important advantage of using a gel dosimetry system is the availability of the 3D dosimetric information without the need to perform additional set up or experimentation. In addition to the 3D dosimetric data, we were able to obtain a complete 3D spatial information using the gel dosimeter. Our results indicated that the virtual cone treatment delivery in the current study was accurate to within 0.94 mm in the end‐to‐end spatial accuracy verification using the anthropomorphic head phantom. Our 3D printed anthropomorphic phantom is a low‐cost SRS phantom (raw material cost < 900 USD), which could be manufactured at any center with 3D printing capabilities (Figure 2). The spatial accuracy result (0.94 mm) in our work is inferior to the original UAB study (0.3 mm mean offset), where a geometric quality assurance was performed with the Winston–Lutz procedure.^16^ The 3D COM distance observed in the gel dosimetric analysis was calculated as 0.66 mm and our MV‐KV imager offset was found to be approximately 0.29 mm (Daily Machine Performance Check). The 0.66 mm distance observed in the 3D dosimetric study, inherently represents the baseline accuracy and limitation of our workflow due to the CBCT slice thickness resolution. Therefore, the true estimate of the geometric accuracy of the SRS virtual cone delivery may be less than 0.94 mm based on the results from the two studies and our expectation. Despite the intrinsic limitation of our CBCT based gel dosimetry workflow, this type of end‐to‐end spatial accuracy analysis is currently only widely achievable with the CBCT imaging tool.

Although the gel dosimeter inherently exhibits a high spatial resolution, the resolution of the image readout technique is and can be a limiting factor in our current gel dosimetry workflow. Our highest attainable spatial resolution with the CBCT modality is achieved by 0.5 mm by 0.5 mm by 1 mm in axial and slice thickness direction, respectively. Therefore, when measuring a small dose region of 5 mm, our workflow is limited by the voxel spacing, particularly by the partial volume artifacts in the slice thickness direction. This is evident in Figure 3c, where we observe a truncated profile in gel due to the partial volume effect in the SP. The low resolution in slice thickness direction which resulted in the partial volume effect, also may have influenced our entire end‐to‐end spatial accuracy analysis. Utilizing an alternative readout method such as CT, may provide a higher spatial resolution, however, a slight image/dosimetric registration error may cause significant uncertainties in the dosimetric results in this type of small‐field dosimetry. Therefore, our study utilized a CBCT imaging protocol, which eliminates the user setup error, and concerns/issues associated with an accurate dosimetric registration. Furthermore, the optimized CBCT image acquisition parameters combined with noise removing algorithms led to an adequate image quality for dosimetric analysis. It is also important to note that the 3D gel dosimetry with the same frame of reference in delivery and readout yielding a high spatial precision, is only achievable with CBCT imaging.

For a comprehensive virtual cone commissioning, absolute dose measurements such as, with ion chamber dosimeter, and an independent dosimetry check with film dosimeter is essential. Although not reported in our paper, we performed absolute dose measurements with the cc13 ionization chamber (IBA Dosimetry Group, Herndon, Virginia, USA) and MicroDiamond detector (PTW, Freiburg, Germany) with our clinical Eclipse v15.6 beam model. The cc13 ion chamber and MicroDiamond detector measurements reported an under response and over response in our dose measurements, corroborating with its respective correction factors (cc13 with 1.15 and MicroDiamond with 0.96 at 4 mm, respectively).^24^ A complete clinical commissioning report necessitates rigorous absolute dose measurements and independent dose check with GafChromic film (Ashland Advanced Materials, Bridgewater, New Jersey, USA) dosimeters. Furthermore, a comprehensive comparison between the gel results against the ion chamber/GafChromic film measurements is valuable to demonstrate the dosimetric accuracy of our gel workflow.

Two different gel calibration processes were utilized in this study. In the 3D gel experiment, a calibration region was utilized to extract the dose response curve in order to calibrate the verification region. This was done specifically to evaluate the relative dosimetric results of our virtual cone method. On contrary, a self‐calibration technique based on one verification slice, was utilized to calibrate the 3D gel data in the end‐to‐end spatial accuracy evaluation. This was only done in order to obtain the spatial accuracy information. An exclusive calibration plan on the skull phantom experiment was not implemented as our goal in this experiment was to treat the skull phantom as a real patient. Furthermore, delivering a calibration plan in another section of the skull phantom requires a couch shift, which is not typically done in this treatment delivery. This also may introduce setup inaccuracies due to the couch shift/movement prior to the dose readout.

An additional research direction may also include improving the slice thickness resolution of our CBCT images. This could potentially be achieved by reconstructing the CBCT images from the raw data at thinner slices comparable to the inplane resolution (0.5 mm). Clinical implementation of Eclipse v18 (with enhanced leaf modeling) is also an area of interest. Furthermore, the use of Ethos (Varian Medical System, Palo Alto, USA) and HyperSight imaging panel could potentially improve the image quality of the gel images (with faster acquisition speed) compared to the Truebeam machine utilized in our study (approximately 6 min per CBCT scan with our current protocol).

Our clinical prescription for treating TN with radiosurgery is based on delivering 80 Gy to the isocenter, with the 50% isodose volume measuring 5 mm in diameter. The 50% isodose volume is placed close to the critical structure that is, brainstem, hence the determination of spatial accuracy is essential in an overall clinical commissioning process. Our current work demonstrated that the gel dosimetry technique employed here measured the 50% isodose width of 5.15 mm (mean of three dose profiles). Furthermore, our study evaluated an end‐to‐end spatial accuracy of the virtual cone delivery, which indicated a sub‐millimeter (0.94 mm) geometric precision.

## 5 CONCLUSION

Verification of a small‐field SRS radiotherapy technique such as virtual cone method presents some unique and complex challenges both in terms of dosimetry and spatial accuracy uncertainties. Additionally, the treatment isocenter is typically placed such that 50% isodose surface is close to a critical structure (brainstem), which necessitates a sub‐millimeter geometric accuracy in the treatment delivery process. In this work, we utilized a relatively novel gel dosimetry tool to verify a virtual cone delivery method. The 50% isodose width of the gel measured 5.56, 5.65, 4.23 mm in axial (AP), axial (LR), SP plane, respectively. The end‐to‐end spatial accuracy analysis, with 3D anthropomorphic phantom, showed that we were able to achieve a 0.94 mm accuracy in the virtual treatment delivery, based on the Euclidean distance between the two centers of masses of the 50% isodose region. The analysis included a CBCT setup error with kV‐MV isocenter uncertainty of 0.29 mm. This type of end‐to‐end spatial accuracy test is only achievable with a gel dosimetry using CBCT readout and the spatial information obtained from this type of SRS technique is valuable to our clinic.

## AUTHOR CONTRIBUTIONS

Tenzin Kunkyab performed experiments/data collection, methodology, interpretation, analysis, project coordinator, manuscript writing. Michael Lamey created the in‐house C# code for the virtual cone technique, manuscript revision. Andrew Jirasek assisted in analysis, interpretation, funding acquisition, manuscript revision. Michael Kudla assisted in 3D printing, manuscript revision. Nathan Becker performed experiments, output verification/second checked virtual cone treatment plans, manuscript revision. Benjamin Mou assisted in conceptualization, clinical guidance, manuscript revision. Derek Hyde (principal investigator) assisted in conceptualization, funding acquisition, interpretation, manuscript revision, supervising the author.

## CONFLICT OF INTEREST STATEMENT

None except Dr. Kunkyab is currently employed by the Mount Sinai Hospital in New York.

## Supporting information



Supporting Information

## Data Availability

The head and neck patient CT data was downloaded from HEAD‐NECK‐RADIOMICS‐HN1 [https://doi.org/10.1038/ncomms5006]. The data was downloaded before the change in their usage policy in 2020.
