# Supplementary material for: Evaluation of dosimetric and spatial accuracy of a virtual cone technique for radiosurgery using linac‐integrated CBCT‐based polymer gel dosimetry
Source: J Appl Clin Med Phys. 2025 Mar 18;26(6):e70081. doi: 10.1002/acm2.70081 (PMC12148801; doi:10.1002/acm2.70081)
Supplement: Supplementary file 1 — Supporting Information [file ACM2-26-e70081-s001.docx]

Appendix A


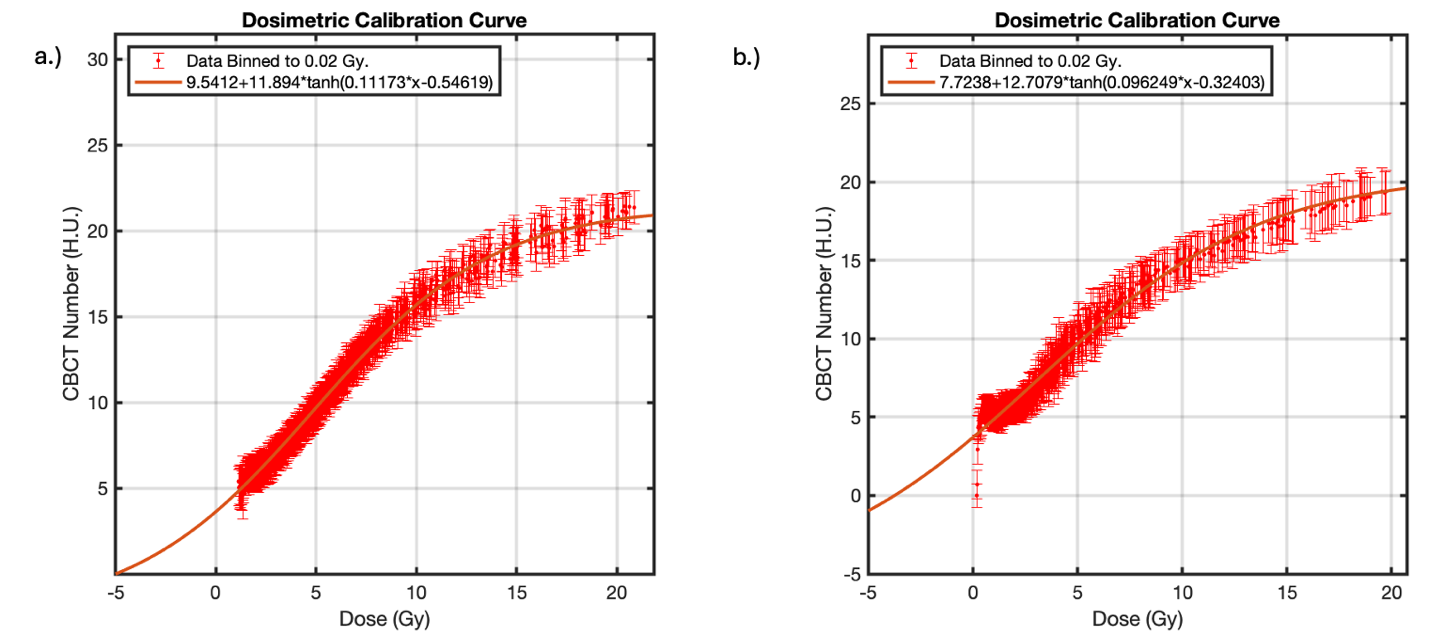


Figure A 1 Illustrates the calibration curve and its parameters of the 3D gel dosimetric analysis (Left) and 3D anthropomorphic gel (Right) experiment.

Appendix B

Table B 1. Presents the calibration parameters for the two gel experiments with 95% confidence interval.

|  | $\alpha$ | $\beta$ | $\gamma$ | $\varphi$ |
| --- | --- | --- | --- | --- |
| 3D gel dosimetric analysis | 9.54 (9.11, 9.98) | 11.89 (11.30,12.48) | 0.11 (0.11,0.12) | 0.55 (0.48,0.61) |
| End-to-end analysis | 7.40 (6.19,8.61) | 13.15 (11.73,14.57) | 0.09 (0.08,0.10) | 0.29 (0.17,0.41) |
